# Comprehend, Cope and Connect: a trauma-informed holistic approach to address mental health crisis as a team

**DOI:** 10.1192/bjb.2025.29

**Published:** 2026-02

**Authors:** Joseph Harris, Isabel Clarke, Pedro Aniceto, Lydia Baxter, Marisol Cavieres, Helen Courtney, Catherine Donaldson, Mark Draper, Alison Farr, Kathryn Gaussen, Elke Haworth, Rose Hooper, Holly Jones, Hasina Khatun, Lorna Lennon, Matthew McNaught, Hazel Nicholls, Katherine Pearson, Jodi Pitt, Megan Wilkinson-Tough, Davina Wong, Simon Riches

**Affiliations:** 1 South London and Maudsley NHS Foundation Trust, London, UK; 2 Southern Health NHS Foundation Trust, Southampton, UK; 3 Dorset Healthcare University Foundation Trust, Poole, UK; 4 Avon and Wiltshire Mental Health Partnership NHS Trust, Salisbury, UK; 5 Solent NHS Trust, Portsmouth, UK; 6 Belfast Health and Social Care Trust, Belfast, UK; 7 Isle of Wight NHS Trust, Newport, UK; 8 Pennine Care NHS Foundation Trust, Stockport, UK; 9 East London NHS Foundation Trust, Bedford, UK; 10 Devon Partnership NHS Trust, Barnstaple, UK; 11 Avon and Wiltshire Mental Health NHS Foundation Trust, Bristol, UK; 12 Institute of Psychiatry, Psychology & Neuroscience, King’s College London, London, UK

**Keywords:** Mental illness, psychological therapy, in-patient services, transdiagnostic, cross-cultural

## Abstract

Comprehend, Cope and Connect (CCC) is a trauma-informed, transdiagnostic and evidence-based psychological intervention for mental health crises that can be applied cross-culturally. CCC has been implemented in acute and crisis mental health settings across the South of England and in services elsewhere in the UK. More recently, it has been taken up and adapted for specialist community settings, including perinatal services, addiction services and primary care settings. A continuously growing evidence base indicates that CCC could be the next step towards solving the national problem of mental health crises. It is now time for CCC to be piloted and researched nationally.

Comprehend, Cope and Connect (CCC) is an evidence-based, trauma-informed, transdiagnostic and cross-cultural psychological intervention designed to meet the needs of individuals in in-patient and other mental health settings, which has been extensively piloted in the South of England.^1,2^ CCC starts with a formulation: a way of making sense of mental health breakdown that encapsulates the individual’s circumstances, strengths and the unique maintaining factors for a mental health crisis in simple terms – and leads to new ways of coping for the individual. This formulation is either worked out collaboratively with the individual (co-produced) or provides a framework for a team to think together and make sense of the crisis. Where the formulation is co-produced, it is understood and owned by the individual but supported by the whole service. If it is a team formulation, it can inform the treatment plan for the service.

Growing evidence from empirical research, primarily conducted in the South of England, indicates that a CCC approach should be implemented more widely in acute psychiatric services across the UK.^3–12^ Therefore, the aim of this article is to emphasise the benefits of implementing the CCC approach more widely across the country. Indeed, patients and mental health professionals have highlighted the need for more psychological interventions in acute and crisis mental health services,^13^ which is further outlined by the National Institute for Health and Care Excellence^14,15^ and in the NHS Long-Term Plan.^16^

## Comprehend, Cope and Connect – a national approach to a national problem

Mental health crises are a significant problem for individuals, families and services across society. This is indicated through the subjective impact of crises (e.g. reduced quality of life, increased carer burden, reduced staff compassion and staff burnout),^17–20^ clinical and risk outcomes (e.g. increase in symptoms of mental illness, risk to self and risk to others)^21,22^ and economic impact (e.g. cost of service provision, loss of productivity).^23^ Unfortunately, this national problem is being steadily exacerbated by ongoing societal stressors, such as the UK cost-of-living crisis^24^ and the fallout from the COVID-19 pandemic.^25^ These pressures have a disproportionate impact on people from marginalised groups who are experiencing additional stressors such as racism,^26^ homophobia,^27^ transphobia^28^ and multiple exclusion homelessness.^29^ In response to these pressures, there are calls for the enhancement of current approaches.^30–32^

## Development of the CCC approach

The second author (I.C.) took the opportunity, as lead psychologist in a then-new National Health Service (NHS) acute in-patient unit, to develop and pilot the CCC approach^1,2^ between 2004 and 2012 to meet precisely these challenges.^11,12^ The consequent publications and recognised success of this initiative led to its extension across four acute services in a large NHS trust^9^ and its subsequent embedding in acute and other services in ten other NHS trusts, predominantly in the South of England.^8,33^ The approach itself has remained largely as originally developed, with the important addition to the formulation diagram of a prominent role for strength and spiritual connection, which arose from cross-cultural collaboration.^6^ Evidence indicates that this trauma-informed and person-centred approach may be the next step in solving the problem of mental health crisis.

## Understanding crisis the CCC way

**Fig. 1:**
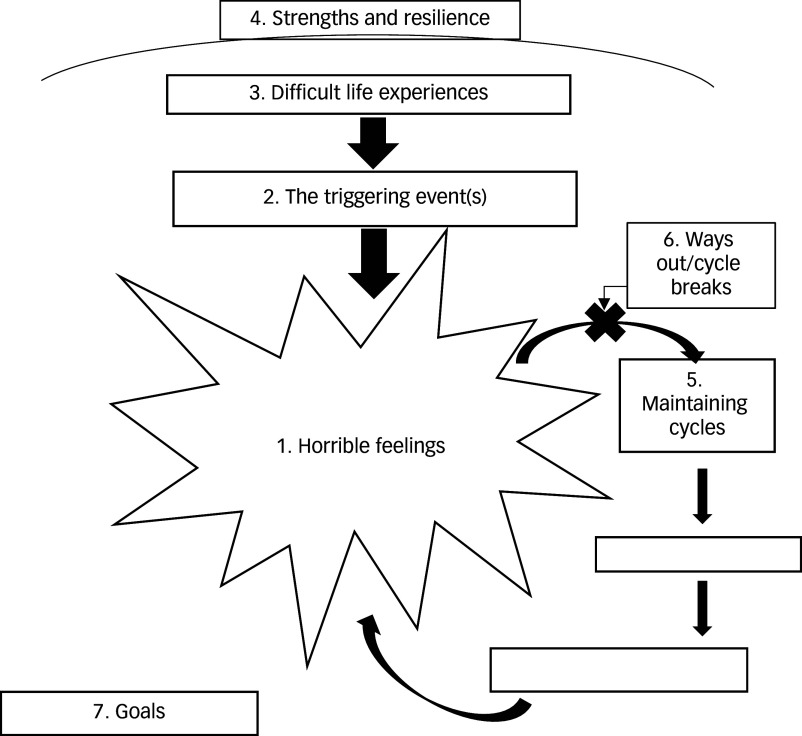
Template for the ‘Comprehend, Cope and Connect’ formulation.

Within the CCC framework, mental health crisis is seen as arising when current stressors, combined with past trauma, lead to the individual’s coping resources becoming overwhelmed. A simple explanation for this interaction is informed by the organisation of human neural circuitry, as delineated in the interacting cognitive subsystems (ICS) model of cognitive architecture.^34,35^ The CCC formulation (Fig. 1) illustrates how current attempts at coping with this interaction (e.g. substance use, dissociation) are understandable yet perpetuate the crisis.

As shown in Fig. 1, the CCC formulation understands the crisis state of ‘horrible feelings’ (1) as brought on by a precipitating event (2) that triggers previous trauma (3). CCC prioritises strengths to increase motivation (4) and draws on these to change the unhelpful coping that maintains the crisis state (5), leading naturally to the identification of alternative coping (6). These changes are used to identify behavioural goals that can be owned by the individual, incorporated into the multidisciplinary team care plan and then supported by the entire staff group, along with family, carers and community supporters (7). Both the necessary behaviour change and the rationale for it can be made clear and evident to all. Training, reflective practice, case discussion and co-working ensure that the individual (or team) trauma-informed formulation is linked to the practice of the entire service, thus promoting a psychologically informed approach and circumventing the disconnect that can exist between therapeutic services and the rest of an organisation. All admissions to acute care can be viewed by the team through the CCC lens, but co-produced individual formulations will be suitable for those who are able to engage and where resources enable this. CCC has been shown to complement the routine responsibilities of psychiatric wards, ensuring safety and effective pharmacological interventions through strengthening engagement between the individual and the team, thus enhancing treatment adherence.

Furthermore, CCC influences the whole milieu by linking an individually tailored approach to communication across the service, with interventions to break perpetuating cycles (such as arousal management, self-compassion) deliverable by all mental health staff after brief training and with ongoing supervision. It further disseminates a compassionate and psychological understanding of crisis. In our clinical opinion, it involves the wider staff group, develops their skills, increases job satisfaction, mitigates against risk of compassion fatigue and supports staff retention.

## Evidence for the approach

Since its initial pilot,^12^ CCC has been introduced into numerous acute services, developed for other services (e.g. community mental health teams, psychotherapy services) and introduced into some primary care networks. The ten acute NHS mental health trusts that have received training and embedded the approach have opted to join the new CCC Research Network, a commitment that further evidences the utility of CCC and the feasibility of embedding it in routine services. Importantly, CCC is empirically supported by a continuously growing evidence base. With a large sample of 131 patients in Hampshire, Araci & Clarke^9^ provided quantitative evidence of both efficacy, with pre- and post- measures showing improvement, and feasibility, with delivery of CCC interventions by staff from both psychological and non-psychological professions. Expanding on this, Riches et al^3^ provided evidence of its impact on and acceptability to acute and crisis staff. In Bristol, Wasiewska et al^4^ provided evidence that when CCC is utilised as a team formulation model in an in-patient setting, it has a positive effect on staff perceptions of patients, subsequent care provided, staff confidence and motivation. In addition, a feasibility study in Lothian,^33^ conducted before the label CCC had been adopted, of 96 patients in in-patient settings showed promising results and also highlighted factors that would be important to consider when implementing and evaluating a whole-service approach. The South London and Maudsley NHS Foundation Trust has produced the rest of the formal evidence to date: Bullock et al^8^ reported that CCC resulted in significant improvements in mood and goal-related outcomes in patients, and was both acceptable and feasible to deliver; Riches et al^36^ reported that CCC is feasible and acceptable to deliver to patients as a remote video call-based therapy, with positive quantitative and qualitative outcomes; and qualitative research undertaken by Harris et al^5^ found that CCC is viewed favourably by patients, carers and multidisciplinary staff. More data is being collected in other services across the UK, and in Pakistan, and with the recent organisation of the CCC Research Network, future research will provide further evidence of the impact CCC is currently having in routine practice. In particular, Harris et al^5^ brought patient and carer voices into the foreground: both patients and carers perceived the CCC model as seeing the whole person, beyond labels, while bringing clarity to crisis and highlighting the important role of emotion. Additionally, patients and carers highlighted what they believed to be important for staff being trained in the CCC approach, emphasising that compassion, empathy and a need to understand the patient experience are priority.

A parallel development has applied CCC to the challenge of complexity in NHS psychotherapy services, and a pilot project has evaluated its application to therapy for people from non-Western cultures in Southern Health NHS Foundation Trust.^6^ CCC was found to be suitable for such adaptation, being less individualistic than much Western therapy, facilitating involvement of family and community, and incorporating faith and spirituality.

This research indicates that CCC warrants further piloting and clinical research across the UK because of its ability to embed routine holistic, trauma-informed practice into acute settings, to engage individuals as partners in their own recovery and to create clear routes to that recovery that can be communicated across the care pathway. It demystifies the psychological approach by breaking down interventions into straightforward packages that can be delivered by a wider spectrum of staff, thus increasing their availability in a context of restricted psychology resources. Indeed, staff have expressed that, with the right support, they would feel confident to deliver CCC interventions and integrate these into standard care planning procedures.^5^ Members of the CCC Research Network are involved in further grass roots research and evaluation projects, with further inclusion of the patient and carer perspective, which is needed to be brought into the CCC literature more deeply.

## CCC across the UK

In line with this, CCC has already successfully been taken up by many different services across the UK. Services in the South East, South West, East and North West of England, and services in Ireland, are all supporting patients and staff teams the ‘Comprehend, Cope and Connect’ way. The following accounts were contributed by members of the CCC Research Network, set up by the first (J.H.) and last (S.R.) authors in 2023, and which now has over 70 members.

### South East England

Southern Health NHS Foundation Trust has embedded CCC throughout its care pathways to operationalise the trust’s strategic objective for trauma-informed working. Here, CCC is the foundation for the overarching trauma-informed approach. For instance, in Southampton’s Acute and Crisis Mental Health Pathway, CCC is the model used for psychological formulations, therapy groups and brief interventions. With nursing and senior management, a new care plan has been developed that incorporates CCC, offering collaborative conversations about distress, risk and safety. This is being piloted in a psychiatric intensive care unit (PICU), with good initial outcomes; individuals report feeling more heard, understood and supported. The Isle of Wight NHS Trust has also implemented CCC across its acute and community services. Formulations are developed with individuals on wards, under the home treatment team and within the community mental health team, with individuals sharing formulations with significant others and keyworkers to ensure ongoing support. In Solent NHS Trust, Portsmouth’s Acute Care Pathway has embedded CCC on its wards: CCC groups are accessed across the pathway, CCC formulations facilitate multidisciplinary team working and inform care planning, CCC treatment is delivered in the community and a CCC passport follows individuals through the system to reduce repeated assessments. The Solent community mental health team service also uses CCC as a ‘foundation intervention’; here, it is effective for mapping out an individual’s distress and how they have become stuck in unhelpful ways of coping. In the Portsmouth and South East Hampshire Older Persons Mental Health Service, in-patient staff value the CCC formulation to better understand individuals and increase staff compassion.

In South London and Maudsley NHS Foundation Trust’s acute in-patient services, CCC has been used in individual psychology sessions to support individuals in making sense of their crises and learning more helpful coping, with support from staff teams to work towards behavioural goals. Here, CCC was adapted into a single-session intervention to suit short ward stays and brief contacts with the home treatment team,^8^ adapted for video call delivery during the COVID-19 pandemic,^36^ and a co-produced training for multidisciplinary staff was also developed.^5^

In South West Hampshire, CCC training is being rolled out across the community mental health teams, to support staff in using CCC formulations with all patients and learning basic intervention skills to help break identified cycles. In the newly formed primary care mental health teams, CCC is used across Southampton’s Enhanced Mental Health Primary Care Team. CCC formulation sessions are offered as an intervention and preparatory step before entering individual or group programmes. The approach is also employed in Hampshire primary care networks: for instance, in Totton it is the overarching approach that guides brief psychological interventions. CCC formulations have facilitated team-working, helping to coordinate support with peer support workers, well-being assessors and social prescribers.

More specialist secondary care services have also adopted CCC in Hampshire. For example, the Hampshire Perinatal Team uses CCC to train mother and baby unit in-patient staff and community perinatal staff, to help build relationships with parents, work collaboratively, and identify and break maintenance cycles. In Southern Health’s specialist Homeless Healthcare Service, CCC has been implemented through supervision and team formulation when thinking about individuals who the team may be unable to progress with, eliciting their strengths and providing a CCC-informed shared language in the team.

### South West England

In the South West, Avon and Wiltshire Mental Health Partnership NHS Trust’s Bristol in-patient services use the CCC formulation model across all nine of their acute, PICU and rehabilitation wards – both in team formulation and in individual sessions. This has proved to be an effective and accessible way of understanding the crisis and it allows further communication when the individual is transferred between wards or is discharged. Additionally, within the Acute Care service in Wiltshire, staff experience of PICU CCC team formulation sessions has been evaluated over the past year using the Team Formulation Quality Scale; initial results indicate that particular strengths of the team formulation are that it is collaboratively developed, key problems and needs are elicited, and there is exploration of significant life events and social and cultural aspects of individuals. In the Devon Partnership NHS Trust’s North Devon Urgent and Inpatient Care Services, CCC guides multidisciplinary team formulations and individual psychology work across adult acute in-patient and rehabilitation units. In University Hospitals Dorset NHS Foundation Trust’s Acute Inpatient Service, the CCC formulation serves as a cornerstone for guiding interventions across the wards, along with offering recommendations to the community mental health teams on discharge, ensuring a seamless transition process.

### Other areas and services

On the borderline between primary and secondary care, CCC has been beneficial in providing a tool and a common language in the East London NHS Foundation Trust’s Bedfordshire Talking Therapies Service. CCC is helping to join up psychotherapy services with community mental health teams, and the benefits of collaboratively working across the system are already being reaped. In the North West, Pennine Care NHS Foundation Trust has been using CCC as the formulation model in Stockport’s in-patient wards for the past 6 years. More recently, community mental health teams (including drug and alcohol services) have adopted the model for use in team formulations and direct joint work with care coordinators, creating a shared language across the Stockport Care Hub. In Ireland, Belfast Health and Social Care Trust’s Acute Inpatient Mental Health Services use the formulation component of CCC as part of their assessment process.

Clearly, CCC has been – and continues to be – innovating mental health services across the UK. Both empirical evidence and the clinical impact in a wide range of acute, in-patient, community and specialist services indicate that piloting CCC on a national level is the next step.

## Overcoming challenges to implementation

Implementation of CCC for a national pilot would be relatively light on resources. Dissemination of CCC within routine practice is achieved by 1- or 2-day training for the whole staff group, with additional training for the therapist group providing support and team formulation.^10^ In the spirit of co-production, Harris et al^5^ provide perspectives from patients, carers and staff on what this training should involve. CCC formulations and interventions can be fitted into the regular work of the ward or service as it complements routine care, leading to a trauma-aware and compassionate workforce. Although there are challenges with embedding any approach across a service, including lack of appropriate resources and training, Harris et al^5^ provide additional staff-generated recommendations for successful implementation of CCC. These include embedding CCC into the culture of a service, integrating CCC into standard care planning procedures, and support from senior staff.

## Conclusion and recommendations

Overall, there is a need for a transdiagnostic, psychological, trauma-informed approach in acute and crisis mental health services. This has been emphasised repeatedly by patients, carers and professionals alike. Mental health crisis is a national issue and deserves a national solution. So far, CCC has been richly piloted in a diversity of services in the South of England and has begun to expand to other regions of the UK. A nationwide approach to this piloting is now warranted. Indeed, CCC is likely to be the next step in overcoming the public health problem of mental health crises, as indicated by a continuously growing evidence base and the widespread uptake of CCC. Helping people in crisis to understand the relevance of their past trauma, how it interacts with current hardship and how their attempts at coping may be what is keeping the crisis state alive, will enhance the good work already being undertaken to support individuals accessing acute mental health services, and other services, who are in distress. To achieve all this, more research is needed, comparing impact across sites, and in particular focusing on the patient experience of the CCC approach. Implementing CCC will open up avenues for greater collaboration between mental health professionals, patients and their families, and offer greater continuity of care assisted by a shared psychologically informed formulation, enabling in-patient and community services to adopt a trauma-sensitive approach. This will likely transform acute wards in the direction of compassion, empathy and understanding, as staff teams and individuals using services join up to overcome crises the Comprehend, Cope and Connect way.

## Data Availability

Data availability is not applicable to this article as no new data were created or analysed in this study.

## About the authors

**Joseph Harris** is a specialist clinical psychologist at South London and Maudsley NHS Foundation Trust, UK. **Isabel Clarke** is a consultant clinical psychologist and clinical lead for the Comprehend, Cope and Connect Programme at Southern Health NHS Foundation Trust, UK. **Pedro Aniceto** is a practitioner psychologist and team coordinator at Dorset Healthcare University Foundation Trust, UK. **Lydia Baxter** is a counselling psychologist at Southern Health NHS Foundation Trust, UK. **Marisol Cavieres** is a lead clinical psychologist for Avon and Wiltshire Mental Health Partnership NHS Trust, UK. **Helen Courtney** is consultant clinical psychologist with the Community Mental Health Teams, Solent NHS Trust, UK. **Catherine Donaldson** is a consultant lead clinical psychologist at Belfast Health and Social Care Trust, UK. **Mark Draper** is a consultant clinical psychologist and acute care lead for psychology and psychological therapies at Southern Health NHS Foundation Trust, UK. **Alison Farr** is a psychological therapist and Comprehend, Cope and Connect lead for Isle of Wight NHS Trust, UK. **Kathryn Gaussen** is a consultant clinical psychologist and community lead for Southern Health NHS Foundation Trust, UK. **Elke Haworth** is a principal clinical psychologist for Southern Health NHS Foundation Trust, UK. **Rose Hooper** is a principal psychologist for Southern Health NHS Foundation Trust, UK. **Holly Jones** is a principal clinical psychologist and clinical lead and service manager for Pennine Care NHS Foundation Trust, UK. **Hasina Khatun** is a consultant clinical psychologist and lead for psychology on the acute care pathway at Solent NHS Trust, UK. **Lorna Lennon** is a clinical service lead for East London NHS Foundation Trust, UK. **Matthew McNaught** is a psychological therapist at Southern Health NHS Foundation Trust, UK. **Hazel Nicholls** is chief psychological professions officer for Southern Health NHS Foundation Trust, UK. **Katherine Pearson** is a clinical psychologist at Devon Partnership NHS Trust, UK. **Jodi Pitt** is a principal clinical psychologist for Southern Health NHS Foundation Trust, UK. **Megan Wilkinson-Tough** is a principal clinical psychologist and head of Bristol Inpatient Psychology for Avon and Wiltshire Mental Health NHS Foundation Trust, UK. **Davina Wong** is a principal clinical psychologist for Southern Health NHS Foundation Trust, UK. **Simon Riches** is a clinical psychologist and lead psychologist for research and development at the South London and Maudsley NHS Foundation Trust and the Institute of Psychiatry, Psychology & Neuroscience, King’s College London, UK.

## References

[1] Clarke I. Meeting Mental Breakdown Mindfully: How to Help the Comprehend, Cope and Connect Way. Routledge, 2021.

[2] Clarke I , Nicholls H. Third-Wave CBT Integration for Individuals and Teams: Comprehend, Cope and Connect. Routledge, 2018.

[3] Riches S , Araci D , Csehi R , Saidel S , Gatherer C , Pierce K , et al. Creating psychologically informed environments on acute psychiatric wards: a lived experience-led study of staff experience. J Psychiatr Intens Care 2024; 20: 35–41.

[4] Wasiewska W , Wilkinson-Tough M , Stephens EJ , Abdi F. Evaluating the impact of team formulation on staff perceptions of patients and impact on care in an acute inpatient setting. J Psychiatr Intens Care 2024; 20: 19–27.

[5] Harris J , Clarke I , Riches S. Developing ‘Comprehend, Cope and Connect’ training for acute and crisis mental health services: staff, patient and carer perspectives. J Psychiatr Intens Care 2023; 19: 33–50.

[6] Phiri P , Clarke I , Baxter L , Yutain Z , Shi K , Yuan X , et al. Evaluation of a culturally adapted cognitive behaviour therapy-based, third-wave therapy manual. World J Psychiatry 2023; 13: 15–35. 36687373 10.5498/wjp.v13.i1.15 PMC9850872

[7] Riches S , Csehi R , Nicholson S , Cohen A , Winter H , Saidel S. Clinical psychologists’ views on facilitating team case formulation in acute and crisis mental health settings. J Psychiatr Intens Care 2024; 15: 43–57.

[8] Bullock J , Whiteley C , Moakes K , Clarke I , Riches S. Single-session Comprehend, Cope and Connect intervention in acute and crisis psychology: a feasibility and acceptability study. Clin Psychol Psychother 2021; 28: 219–25. 32833291 10.1002/cpp.2505

[9] Araci D , Clarke I. Investigating the efficacy of a whole team, psychologically informed, acute mental health service approach. J Mental Health 2016; 26: 307–11. 10.3109/09638237.2016.1139065 26855262

[10] Clarke I. The Emotion Focused Formulation Approach: bridging individual and team formulation. Clin Psychol Forum 2015; 275: 28–32.

[11] Clarke I , Wilson H. Cognitive Behaviour Therapy for Acute Inpatient Mental Health Units: Working with Clients, Staff and the Milieu. Routledge, 2009.

[12] Durrant C , Clarke I , Tolland A , Wilson H. Designing a CBT service for an acute inpatient setting: a pilot evaluation study. Clin Psychol Psychother 2007; 14: 117–25.

[13] Small C , Pistrang N , Huddy V , Williams C. Individual psychological therapy in an acute inpatient setting: service user and psychologist perspectives. Psychol Psychother 2018; 91: 417–33. 29345801 10.1111/papt.12169

[14] National Institute for Health and Care Excellence. Rehabilitation for Adults with Complex Psychosis (NICE Guideline NG181). NICE, 2020. 32991081

[15] National Institute for Health and Care Excellence (NICE). Psychosis and Schizophrenia in Adults: Treatment and Management (NICE Clinical Guideline CG178). NICE, 2014.

[16] NHS England. The NHS Long Term Plan. NHS, 2019 (https://www.longtermplan.nhs.uk/publication/nhs-long-term-plan).

[17] Tane E , Fletcher I , Bensa S. Staff compassion in acute mental health wards: a grounded theory investigation. J Mental Health 2022; 31: 657–65. 10.1080/09638237.2021.1875402 33612064

[18] O’Connor K , Neff D , Pitman S. Burnout in mental health professionals: a systematic review and meta-analysis of prevalence and determinants. Eur Psychiatry 2018; 53: 74–99. 29957371 10.1016/j.eurpsy.2018.06.003

[19] Albert R , Simpson A. Double deprivation: a phenomenological study into the experience of being a carer during a mental health crisis. J Adv Nurs 2015; 71: 2753–62. 26249710 10.1111/jan.12742

[20] Basu D. Quality-of-life issues in mental health care:pPast, present, and future. Geriatr J Psychiatry 2004; 7: 35–43.

[21] Penterman B , Nijman H. Assessing aggression risks in patients of the ambulatory mental health crisis team. Commun Mental Health J 2011; 47: 463–71. 10.1007/s10597-010-9348-7 20886294

[22] Bowers L , Banda T , Nijman H. Suicide inside: a systematic review of inpatient suicides. J Nerv Mental Dis 2010; 198: 315–28. 10.1097/NMD.0b013e3181da47e2 20458192

[23] Knapp M , Wong G. Economics and mental health: the current scenario. World Psychiatry 2020; 19: 3–14. 31922693 10.1002/wps.20692 PMC6953559

[24] Lawson G , Haggar T , Hewlett K , Hall S , Piggott H , Hesketh R , et al. *Experiencing the Cost-Of-Living Crisis: The Impact on Mental Health*. King’s College London, 2023 (10.18742/pub01-154).

[25] Anaya L , Howley P , Waqas M , Yalonetzky G. Locked down in distress: a quasi-experimental estimation of the mental health fallout from the COVID-19 pandemic. Econ Inquiry 2023; 62: 56–73.

[26] Paradies Y , Ben J , Denson N , Elias A , Priest N , Pieterse A , et al. Racism as a determinant of health: a systematic review and meta-analysis. PLoS One 2015; 10: e0138511. 26398658 10.1371/journal.pone.0138511 PMC4580597

[27] Ventriglio A , Castaldelli-Maia J , Torales J , De Berardis D , Bhugra D. Homophobia and mental health: a scourge of modern era. Epidemiol Psychiatr Sci 2021; 30: e52. 10.1017/S2045796021000391 PMC8264802 34185635

[28] Price M , Hollinsaid N , McKetta S , Mellen E , Rakhilin M. Structural transphobia is associated with psychological distress and suicidality in a large national sample of transgender adults. Soc Psychiatry Psychiatr Epidemiol 2024; 59: 285–94. 37165214 10.1007/s00127-023-02482-4 PMC10171731

[29] Pattison B , McCarthy L. The role of mental health in multiple exclusion homelessness. Soc Policy Soc 2020; 21: 405–21.

[30] Huber J , Milton A , Brewer M , Norrie L , Hartog S , Glozier N. The effectiveness of brief non-pharmacological interventions in emergency departments and psychiatric inpatient units for people in crisis: a systematic review and narrative synthesis. Austr N Z J Psychiatry 2024; 58: 207–26. 10.1177/00048674231216348 38140961

[31] Alang S , McAlpine D. Pathways to mental health services and perceptions about the effectiveness of treatment. Soc Mental Health 2019; 9: 388–407.

[32] Correll C. Acute and long-term adverse effects of antipsychotics. CNS Spectr 2007; 12: 10–4. 10.1017/s1092852900015959 18389927

[33] Paterson C , Karatzias T , Dickson A , Harper S , Dougall N , Hutton P. Psychological therapy for inpatients receiving acute mental health care: a systematic review and meta-analysis of controlled trials. Br J Clin Psychol 2018; 57: 453–72. 29660770 10.1111/bjc.12182

[34] Clarke I. Cognitive therapy and serious mental illness: an interacting cognitive subsystems approach. Clin Psychol Psychother 1999; 6: 375–83.

[35] Teasdale JD , Barnard PJ. Affect, Cognition and Change: Remodelling Depressive Thought. Lawrence Erlbaum Associates, 1993.

[36] Riches S , Csehi R , Steer N , Azevedo L , Vasile R , Lokhande M. Video call-based psychological therapy for inpatients during the COVID-19 lockdown. Cyberpsychol Bull 2020; 3.

